# Blood-Cerebrospinal Fluid Barrier Gradients in Mild Cognitive Impairment and Alzheimer's Disease: Relationship to Inflammatory Cytokines and Chemokines

**DOI:** 10.3389/fnagi.2018.00245

**Published:** 2018-08-21

**Authors:** Brian R. Ott, Richard N. Jones, Lori A. Daiello, Suzanne M. de la Monte, Edward G. Stopa, Conrad E. Johanson, Charles Denby, Paula Grammas

**Affiliations:** ^1^Department of Neurology, Alpert Medical School of Brown University, Rhode Island Hospital, Providence, RI, United States; ^2^George & Anne Ryan Institute for Neuroscience, University of Rhode Island, Kingston, RI, United States; ^3^Division of Neuropathology, Department of Pathology, Alpert Medical School of Brown University, Rhode Island Hospital, Providence, RI, United States; ^4^Department of Neurosurgery, Alpert Medical School of Brown University, Rhode Island Hospital, Providence, RI, United States

**Keywords:** blood brain barrier, choroid plexus, inflammation, Alzheimer, mild cognitive impairment (MCI)

## Abstract

**Background:** The pathophysiology underlying altered blood-cerebrospinal fluid barrier (BCSFB) function in Alzheimer's disease (AD) is unknown but may relate to endothelial cell activation and cytokine mediated inflammation.

**Methods:** Cerebrospinal fluid (CSF) and peripheral blood were concurrently collected from cognitively healthy controls (*N* = 21) and patients with mild cognitive impairment (MCI) (*N* = 8) or AD (*N* = 11). The paired serum and CSF samples were assayed for a panel of cytokines, chemokines, and related trophic factors using multiplex ELISAs. Dominance analysis models were conducted to determine the relative importance of the inflammatory factors in relationship to BCSFB permeability, as measured by CSF/serum ratios for urea, creatinine, and albumin.

**Results:** BCSFB disruption to urea, a small molecule distributed by passive diffusion, had a full model coefficient of determination (*r*^2^) = 0.35, and large standardized dominance weights (>0.1) for monocyte chemoattractant protein-1, interleukin (IL)-15, IL-1rα, and IL-2 in serum. BCSFB disruption to creatinine, a larger molecule governed by active transport, had a full model *r*^2^ = 0.78, and large standardized dominance weights for monocyte inhibitor protein-1b in CSF and tumor necrosis factor-α in serum. BCSFB disruption to albumin, a much larger molecule, had a full model *r*^2^ = 0.62, and large standardized dominance weights for IL-17a, interferon-gamma, IL-2, and VEGF in CSF, as well IL-4 in serum.

**Conclusions:** Inflammatory proteins have been widely documented in the AD brain. The results of the current study suggest that changes in BCSFB function resulting in altered permeability and transport are related to expression of specific inflammatory proteins, and that the shifting distribution of these proteins from serum to CSF in AD and MCI is correlated with more severe perturbations in BCSFB function.

## Introduction

Blood-brain barrier (BBB)permeability increases with normal aging and may be an important mechanism in the initiation or worsening of cerebral microvascular disease leading to dementia (Farrall and Wardlaw, 2009). Vascular dysfunction has a critical role in Alzheimer's disease (AD), and multiple avenues of investigation point to the essential role of neurovascular and BBB mechanisms as contributors to both onset and progression of AD (Bell and Zlokovic, 2009). Current pathological, clinical, and imaging studies support the existence of a blood-brain barrier (BBB) defect in the pathogenesis of AD (Blennow et al., 1990; Stewart et al., 1992; Kalaria, 1999; Bowman and Quinn, 2008; Farrall and Wardlaw, 2009) that affects passive as well as active transport mechanisms (Kalaria and Harik, 1989a,b; Harik and Kalaria, 1991; Johanson et al., 2018).

*In vivo* BBB studies in humans with AD have used measurements of albumin to assess blood-cerebrospinal fluid barrier (BCSFB) integrity as an indicator of BBB disruption. Albumin is a large molecule synthesized predominantly by the liver, so any amounts found in brain or cerebrospinal fluid (CSF) are assumed to be mainly deposited within the extracellular space via the peripheral circulation. Of 10 studies reported in a recent review that examined BBB integrity in AD compared to controls using the “CSF/serum albumin index,” six found evidence of BBB disruption, and four did not (Bowman and Quinn, 2008). A recent study involving rigorous diagnostic criteria for subject selection examined 36 mild to moderate AD subjects and found evidence of an abnormal CSF/albumin index in 22% (Bowman et al., 2007). Age, sex, and apolipoprotein E (ApoE) status did not correlate with the index results. Correlation of the index with measures of disease progression over 1 year (MMSE, Clinical Dementia Rating sum of boxes, and ventricular volume on MRI) suggested a role for BBB disruption as a modifier of the disease (Bowman et al., 2007). Since normal older subjects and subjects with prodromal AD or mild cognitive impairment (MCI) were not assessed, it is not clear whether this observation is an epiphenomenon of established disease or a contributor to the early pathogenesis of AD.

The time point at which this mechanism becomes important in the pathogenesis of AD is unknown. In an experiment with AD model Tg2576 transgenic mice, altered BBB permeability (leakage) preceded neuritic plaque formation suggesting a central role for early structural changes to the BBB caused by elevated A-beta (Ujiie et al., 2003). Two recent studies of subjects who underwent dynamic contrast enhanced magnetic resonance imaging found evidence of disrupted BBB function at the stage of MCI (Montagne et al., 2015; van de Haar et al., 2016). In the latter study, CSF levels of several studied cytokines (interleukins IL-2, IL-6, and IL-8, tumor necrosis factor-α, and interferon-γ) were not correlated with radiologic changes in the BBB. In a 3-year prospective study of older people with and without dementia at baseline, non-demented women at age 85 (*n* = 3) who developed dementia during the follow-up had a higher CSF/serum albumin ratio than those not developing dementia (10.4 ± 2.0 vs. 6.0 ± 1.9; *p* = 0.007), suggesting that BCSFB disruption may occur before the onset of clinical symptoms (Skoog et al., 1998).

We recently reported a study of BCSFB markers in 21 healthy controls compared to 21 patients with cognitive impairment classified as MCI or AD. Dissipating CSF/serum ratios in MCI and AD, toward the equilibrium value of 1.0, suggested disease-associated changes in permeability (urea and albumin) and barrier transporter activity (creatinine/creatine), particularly in those with MCI. Urea finely differentiated altered BCSFB permeability from normal barrier function between patients and controls. CSF/serum ratios were consistent with disrupted BBB function that likely occurs even at the prodromal stage of AD, suggesting that measurement of CSF and serum urea and creatinine may be sensitive novel biomarkers of modified fluid biochemistry and loss of BBB function in MCI and AD (Johanson et al., 2018).

The underlying pathophysiology of this phenomenon remains poorly understood (Viggars et al., 2011). Some genetic mouse models producing A-beta do not support a role of BBB disruption in AD (Poduslo et al., 2001), suggesting that non-genetic processes may be more likely causative of the phenomenon in late onset AD. The “vascular hypothesis,” based on evidence of reduced BBB integrity preceding other AD pathology, holds that BBB leakiness in AD is likely due to hypoxia, ischemia and neuroinflammation (Fiala et al., 2002; Pluta and Amek, 2008) leading to vascular deterioration and apoptosis. However, there is also evidence for deregulated low-density lipoprotein receptor-related protein 1 and receptor for advanced glycation end-products (LRP1/RAGE) mediated transport (Deane and Zlokovic, 2007; Provias and Jeynes, 2014), altered agrin expression (Rauch et al., 2011), modifications in adhesion molecules and leukocyte migration (Zenaro et al., 2016), and neoangiogenesis as inciting factors (Biron et al., 2011). Late in the process, a “traffic jam” at the BBB may promote greater accumulation of A-beta proteins in the cerebral vasculature (Vinters and Pardridge, 1986; Agyare et al., 2013). One study found a gender effect with the BCSFB being more severely affected in men with AD, suggesting potential hormonal factors as well (Algotsson and Winblad, 2007).

The ApoE4 allele has been associated with a higher likelihood of BBB disruption in post mortem samples (Zipser et al., 2007). ApoE4 may predispose to accelerated accumulation of the proinflammatory cytokine cyclophilin A and pericyte loss (Halliday et al., 2016). We have described the relationships between ApoE, A-beta, and the BBB in AD. Alterations in the expression and distribution of the BBB A-beta transporters, LRP1/RAGE, in AD appear to magnify the effects of ApoE4 expression in adversely influencing A-beta burden and BBB permeability (Donahue and Johanson, 2008).

It is well recognized that neuroinflammation is an important component of AD pathophysiology (Halliday et al., 2000; Zhang and Jiang, 2015). Little is known, however, about the potential relationships between inflammation and the BCSFB in the pathogenesis of AD (Takeda et al., 2014). We previously analyzed AD patient and control subjects for inflammatory cytokine/chemokine and related trophic factor expression in blood and CSF using multiplex ELISAs. The findings suggested that in the early stages of neurodegeneration, cognitive impairment may be mediated by systemic plus CNS-derived inflammatory processes, and that the pathogenic processes include inhibition of neuronal survival, neuroprotective, neurotrophic, and angiogenic mechanisms and activation of inflammatory-mediated injury to myelin (De la Monte et al., 2017).

Our current study sought to explore the pathophysiology of disrupted BBB function in AD by directly comparing relationships between changes in BCSFB biomarkers and inflammatory cytokines and chemokines in a mixed sample of subjects with normal cognition, MCI and AD. It is our hypothesis that inflammatory cytokines and chemokines contribute directly to disrupted BCSFB function. We utilized CSF/serum ratios of not only albumin (molecular weight 66,437 Daltons), but also urea (60 Daltons) and creatinine (113 Daltons), in order to explore the potential roles of inflammatory markers in earlier AD pathology when BBB disruption may be related to leakage of small molecules or damage to active transport mechanisms.

## Materials and methods

### Participants

The methods for this study have been previously reported (De la Monte et al., 2017). This cross-sectional study was designed to evaluate inflammatory profiles in prospectively banked paired serum and CSF samples from patients with mild cognitive impairment (MCI) or AD. Lumbar CSF and blood were concurrently collected from cognitively healthy controls (*N* = 21) and patients with MCI (*N* = 8) or AD (*N* = 11). Diagnoses of AD were made according to NINCDS-ADRDA criteria (McKhann et al., 1984), while diagnoses of MCI were made according to MCI International Working Group consensus criteria (Winblad et al., 2004). Exclusion criteria for patients included history of traumatic brain injury, major psychiatric illness other than for treated depression, alcoholism and substance abuse in the past 3 years, clinically significant stroke, hydrocephalus, untreated B12 deficiency or hypothyroidism. Structural brain lesions that could account for MCI or dementia symptoms were excluded by brain MRI prior to lumbar puncture.

The patients were evaluated at the Rhode Island Hospital (RIH) Alzheimer's Disease and Memory Disorders Center between 2010 and 2016. The biological fluid samples were collected in accordance with the Alzheimer's Disease Neuroimaging Initiative protocol. Paired serum and CSF samples were obtained as part of a neurologic diagnostic evaluation or as add-on donations at the time of a clinical trial or observational research study visit. Following collection, the samples were aliquoted into 2 ml sterile polypropylene screw capped tubes and frozen at −80°C.

All subjects signed written informed consent documents approved by the RIH human subjects committee (RIH#210946-17) to allow their serum and CSF samples to be banked for future research.

Control patients were evaluated for headache in the Rhode Island Hospital Emergency Department between October 2014 and December 2015, and the biological fluid samples were collected in accordance with standard hospital practice. The control subjects did not carry a diagnosis of active infection and were free of cognitive and primary neurological disorders other than headache based on review of hospital outpatient, inpatient and emergency department records. The main inclusion criteria for the fluid samples were that sufficient volumes of the paired serum and CSF samples be available for analysis, and that routine assays of CSF, including protein and glucose concentrations, cell counts, and Gram stain results be within normal limits. This study (RIH#633481-13) was approved by the Lifespan Hospitals Institutional Review Board.

### Assays

Direct Binding Enzyme-linked Immunosorbent assay (ELISA): Amyloid-beta peptide of the amyloid precursor protein (Aβ1-42) and phospho-tau (pTau-307) immunoreactivity were measured in direct binding ELISAs. Serum samples diluted 1:100 and CSF diluted 1:4 in Tris-buffered saline (TBS) were adsorbed (50 μl each) to the well bottoms of Maxisorp 96-well plates (Nunc) by overnight incubation at 4°C, then blocked for 3 h at room temperature with 1% bovine serum albumin (BSA) in TBS. After washing, the samples were incubated with primary antibody (0.1–0.4 μg/ml) for 1 h at 37°C. Immunoreactivity was detected with horseradish peroxidase-conjugated secondary antibody and Amplex UltraRed soluble fluorophore. Fluorescence intensity was measured (Ex 565 nm/Em 595 nm) in a SpectraMax M5 microplate reader (Molecular Devices, Sunnyvale, CA).

Multiplex Human Cytokine ELISAs: Bead-based multiplex ELISAs were employed to assess levels of 27 pro-inflammatory cytokines and chemokines and trophic factors in serum and CSF. The list of cytokines, chemokines, and trophic factors, their abbreviations, and functions both systemically and in the central nervous system are summarized in Table 1. Prior to use, thawed serum samples were diluted 1:4 in assay dilution buffer (ADB), whereas CSF was used undiluted. The samples were incubated with the beads according to the manufacturer's protocol. Captured antigens were detected with secondary antibodies and plates were read in a MAGPIX (Bio-Rad, Hercules, CA).

**Table 1:** Serum and CSF cytokines, chemokines, and trophic factors.

**Factor**	**Factor abbreviation**	**Function class**	Systemic actions^*^	CNS actions^*^
Granulocyte colony stimulating factor	G-CSF	Cytokine	Pro-inflammatory	Neuro-protection
Granulocyte macrophage colony stimulating factor	GM-CSF	Cytokine	Pro-inflammatory	Neuro-protection
Interleukin-1 receptor antagonist	IL-1RA	Cytokine	Adhesion	Neuro-protection
Interleukin-1beta	IL-1β	Cytokine	Pro-inflammatory	Pro-inflammatory
Interleukin-2	IL-2	Cytokine	Pro-inflammatory	Neuro-protection
Interleukin-4	IL-4	Cytokine	Anti-Inflammatory	Anti-inflammatory
Interleukin-5	IL-5	Cytokine	Pro-inflammatory	Pro-inflammatory
Interleukin-6	IL-6	Cytokine	Pro-inflammatory	Pro-inflammatory
Interleukin-9	IL-9	Cytokine	Pro-inflammatory	Pro-inflammatory
Interleukin-10	IL-10	Cytokine	Anti-Inflammatory	Neuro-protection
Interleukin-12, p70	IL-12p70	Cytokine	Pro-inflammatory	Pro-injury
Interleukin-13	IL-13	Cytokine	Pro-inflammatory	Pro-injury
Interleukin-15	IL-15	Cytokine	Pro-inflammatory	Pro-inflammatory
Interleukin-17a	IL-17a	Cytokine	Pro-inflammatory	Pro-inflammatory
Tumor necrosis factor-alpha	TNF-α	Cytokine	Pro-inflammatory	Neurodegeneration
Eosinophil chemotactic protein	Eotaxin	Chemokine	Pro-inflammatory	Anti-trophic
Interferon-gamma	IFN-γ	Chemokine	Pro-inflammatory	Pro-inflammatory
Interleukin-8	IL-8	Chemokine	Pro-inflammatory	Pro-injury
Interferon gamma induced protein	IP-10	Chemokine	Pro-inflammatory	Neurodegeneration
Monocyte chemoattractant protein 1	MCP-1	Chemokine	Pro-inflammatory	Neurodegeneration
Macrophage inflammatory protein 1 alpha	MIP-1α	Chemokine	Pro-inflammatory	Neurodegeneration
Macrophage inflammatory protein 1 beta	MIP-1β	Chemokine	Pro-inflammatory	Neurodegeneration
Platelet derived growth factor- bb	PDGF-bb	Chemokine	Pro-inflammatory	Neuroprotection
Regulated upon activation, normal T-cell expressed and secreted	RANTES	Chemokine	Pro-inflammatory	Pro-inflammatory
Basic fibroblast growth factor	b-FGF	Trophic	Angiogenesis	Neurotrophic
Interleukin-7	IL-7	Trophic	Pro-inflammatory	Pro-injury
Vascular endothelial growth factor	VEGF	Trophic	Angiogenesis	Angiogenesis

* *See reference De la Monte, et al for additional information*.

### Statistical analysis

We compared mean differences in expression levels for cytokines, chemokines, and trophic factors between participants with MCI or AD and those without cognitive impairment, and expressed these differences as Cohen's *d* effect sizes (mean difference divided by pooled standard deviation). Conventional interpretation of *d* is that differences of 0.2, 0.5, and 0.8 demarcate small, medium and large effect size differences. Confidence intervals for Cohen's *d* were obtained with reference to the non-central *t* distribution function. The direction of the comparison is such that when *d* is negative, the mean in the cognitively intact group is lower than in the cognitively impaired group. *P*-values derived from two-sample *t*-tests were not corrected for multiple comparisons. With 27 factors, and comparing across serum and CSF, there were 2 × 27 comparisons. The significance level associated with a Bonferroni correction for 54 comparisons is about *p* < 0.001 (0.05/54 = 0.0009).

To evaluate the relative importance of the cytokines, chemokines, and trophic factors to BCSFB ratios for urea, creatinine and albumin, we performed a dominance analysis. Dominance analysis is useful for identifying the relative importance of independent variables and is based on comparisons and combinations of fit statistics from each subset regression (Budescu, 1993). We based our inference regarding the most important independent variable on the general dominance statistic. The general dominance statistic is the average across all possible incremental or marginal contributions an independent variable makes to overall model fit (*R*^2^), i.e., an average of all possible combinations of models including (and not including) a given independent variable. We used this to produce a rank ordering of independent variable importance.

In addition we performed an additive decomposition of the overall model *R*^2^, reflecting the variable's contribution to the model *R*^2^ across all possible models. Because dominance analysis involves fitting all possible subsets of regression equations and this is very computer intensive, we limited our consideration to models with the strongest bivariable association between predictor and outcome. Our criterion for inclusion was that the bivariable association was at least *R*^2^ = 0.1, and we allowed a maximum of 12 predictors per model chosen in order of strength of bivariable association.

## Results

### Demographics of the groups

Demographic and clinical characteristics of the subjects are listed in Table 2. As can be seen in Table 2, the CSF Aβ1-42 /p-tau ratios for the AD and the MCI patients were comparable and differed significantly from the controls. This suggests shared pathology between the AD and MCI groups and is not surprising, since MCI is generally regarded as a transitional stage in the development of AD (Morris et al., 2001). Because of our small patient sample sizes and the identical AD biomarkers in CSF, we combined the AD and MCI subjects for all analyses comparing patients and controls.

**Table 2:** Demographic and clinical characteristics of subjects.

	**AD (*N* = 11)**	**MCI (*N* = 8)**	**Normal (*N* = 21)**
Age (years)	67 (10) [49–83]	69 (7) [59–77]	46 (11) [28–77]
Sex (male/female)	5/6	7/1	12/9
MMSE score	22 (5) [13–28]	26 (3) [21–30]	N.A.
CSF Aβ_1−42_/p-tau ratio	6.03 (0.96) [4.8–7.8]	5.79 (2.3) [0.72–8.01]	8.2 (1.4) [6.03–10.47]
CSF/serum urea ratio	0.85 (0.07 [0.73–0.93]	0.98 (0.18) [0.78–1.34]	0.79 (0.15) [0.53–1.10]
CSF/serum creatinine ratio	1.15 (0.14) [0.93–1.40]	1.13 (0.20) [0.85–1.38]	1.5 (0.4) [0.9–2.2]
CSF/serum albumin ratio	0.66 (0.34) [0.22–1.32]	1.2 (1.8) [0.2–5.1]	0.45 (0.24) [0.15–1.06]

All statistics are mean (standard deviation) [range].

AD, Alzheimer's disease; MCI, mild cognitive impairment.

MMSE, Mini-Mental State Examination.

*CSF, cerebrospinal fluid*.

The CSF/serum ratios for urea and albumin were higher for patients than controls. The ratio was in the opposite direction for creatinine. This difference in direction is due to the BCSFB to urea and albumin being maintained by passive diffusion mechanism, while creatine and creatinine are governed by active transport between blood, brain and CSF (Tachikawa and Hosoya, 2011; Johanson et al., 2018).

Since the cognitively impaired subgroups of AD and MCI were small and comparable in both age as well as A-beta and tau indices of pathology, we combined these two groups for purposes of statistical analysis of group difference assays for the cytokine, chemokine, and trophic factors. Table 3 shows Cohen's *d* effect sizes for the comparison of these factor levels between the cognitively intact and cognitively impaired groups.

**Table 3:** Cohen's d for serum and CSF cytokines, chemokines, and trophic factors in comparisons between subjects with and without cognitive impairment.

	**Serum**			**CSF**		
**Factor**	***d***	**(95% CI)**	***P***	***d***	**(95% CI)**	***p***
VEGF	−0.12	(−0.74, 0.50)	0.71	2.06	(1.24, 2.86)	<0.001
TNFALPHA	−1.23	(−1.91, −0.55)	<0.001	−0.52	(−1.16, 0.12)	0.11
RANTES	−1.30	(−1.98, −0.61)	<0.001	0.15	(−0.48, 0.78)	0.64
PDGFBB	−2.09	(−2.86, −1.30)	<0.001	1.10	(0.41, 1.77)	0.002
MIP1B	−0.11	(−0.73, 0.51)	0.73	−0.42	(−1.05, 0.22)	0.20
MIP1A	−0.97	(−1.63, −0.31)	0.004	−0.40	(−1.04, 0.23)	0.22
MCP1	0.71	(0.06, 1.34)	0.03	0.47	(−0.17, 1.11)	0.15
IP10	0.65	(0.01, 1.29)	0.046	0.33	(−0.31, 0.96)	0.31
IL9	−0.44	(−1.06, 0.19)	0.18	−0.67	(−1.31, −0.02)	0.04
IL8	0.42	(−0.21, 1.05)	0.19	0.27	(−0.37, 0.90)	0.41
IL7	0.16	(−0.46, 0.78)	0.61	−0.69	(−1.33, −0.03)	0.04
IL6	0.59	(−0.04, 1.23)	0.07	0.47	(−0.18, 1.10)	0.16
IL5	−4.33	(−5.47, −3.17)	<0.001	−0.92	(−1.58, −0.25)	0.01
IL4	−2.80	(−3.67, −1.91)	<0.001	−0.52	(−1.16, 0.12)	0.11
IL2	1.59	(0.86, 2.29)	<0.001	−0.23	(−0.86, 0.40)	0.48
IL1RA	0.18	(−0.44, 0.80)	0.58	−0.06	(−0.69, 0.57)	0.86
IL1B	−0.78	(−1.42, −0.13)	0.02	−0.05	(−0.68, 0.58)	0.87
IL17A	−1.71	(−2.43, −0.97)	<0.001	1.03	(0.35, 1.69)	0.003
IL15	1.91	(1.14, 2.65)	<0.001	−0.00	(−0.63, 0.62)	0.99
IL13	−1.00	(−1.65, −0.33)	0.003	−0.97	(−1.63, −0.30)	0.005
IL12P70	0.10	(−0.52, 0.72)	0.76	0.23	(−0.40, 0.86)	0.48
IL10	0.24	(−0.38, 0.86)	0.45	−0.42	(−1.06, 0.22)	0.20
IFNG	0.07	(−0.55, 0.69)	0.82	−0.72	(−1.36, −0.06)	0.03
GMCSF	1.69	(0.95, 2.41)	<0.001	2.47	(1.62, 3.30)	<0.001
GCSF	0.40	(−0.23, 1.02)	0.22	0.30	(−0.34, 0.93)	0.36
EOTAX	−1.20	(−1.87, −0.52)	<0.001	−0.29	(−0.92, 0.34)	0.37
BFGF	0.63	(−0.01, 1.26)	0.06	0.96	(0.28, 1.62)	0.005

*When d is negative, the mean in the cognitively intact group is lower than in the cognitively impaired group*.

### Cytokine, chemokine, and trophic factors predicting BCSFB to urea

The relative dominance of each independent variable was derived by examining its contribution to model *R*^2^ when it was included vs. when it was not included among all possible models.

**Figure 1:**
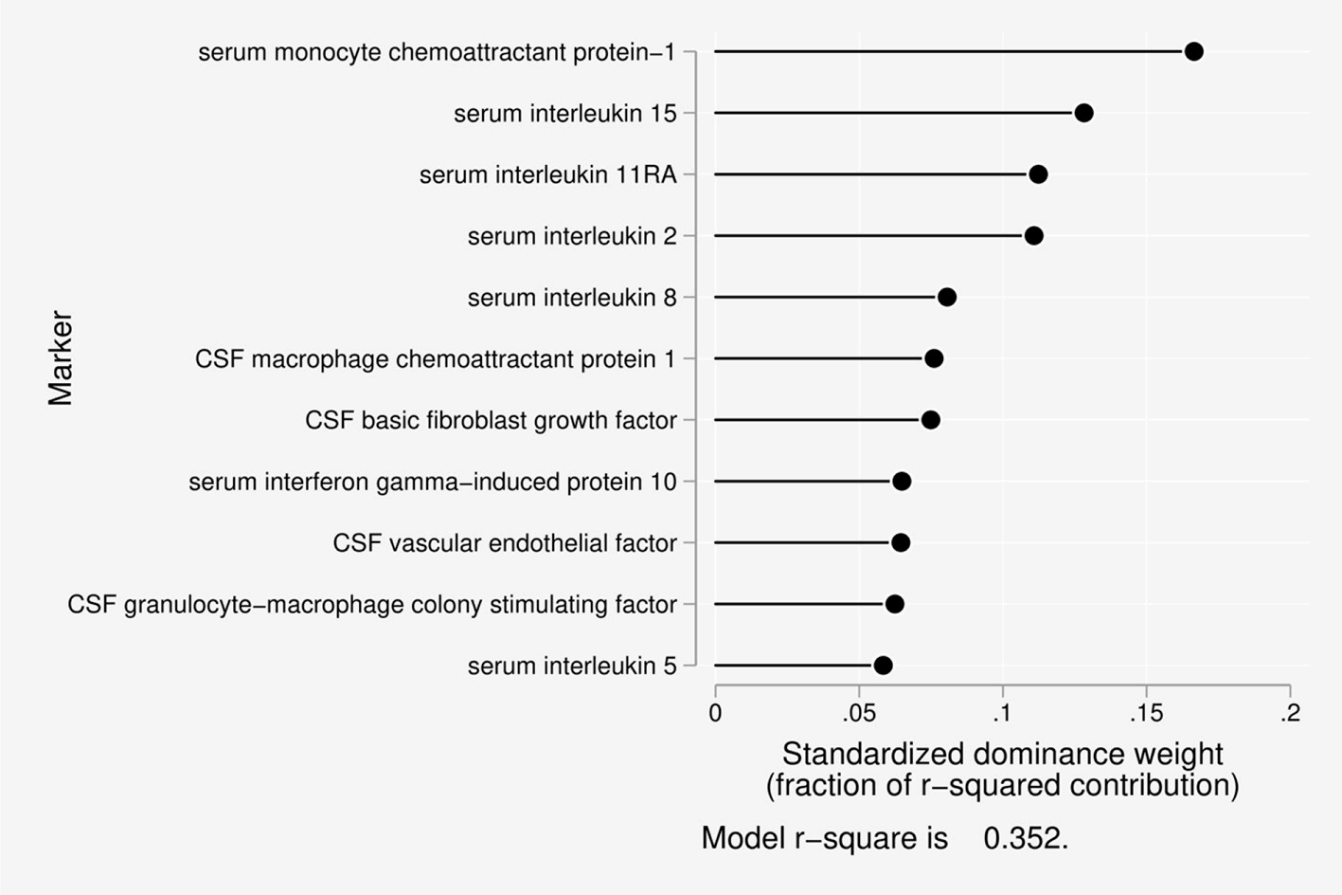
Proportionate contribution of serum and CSF factors to model r-square for BCSFB ratios for urea.

Eleven predictors were included in the dominance analysis for BCSFB permeability to urea that included estimates of 2,047 subset models. The overall model *R*^2^ was 0.35. The proportionate contribution to this model *R*^2^ is shown in Figure 1. The dominant predictor for the urea CSF/serum ratio was serum monocyte chemoattractant protein 1 (MCP-1), which accounted for 17% of the overall model *R*^2^.

### Cytokine, chemokine, and trophic factors predicting BCSFB to creatinine

**Figure 2:**
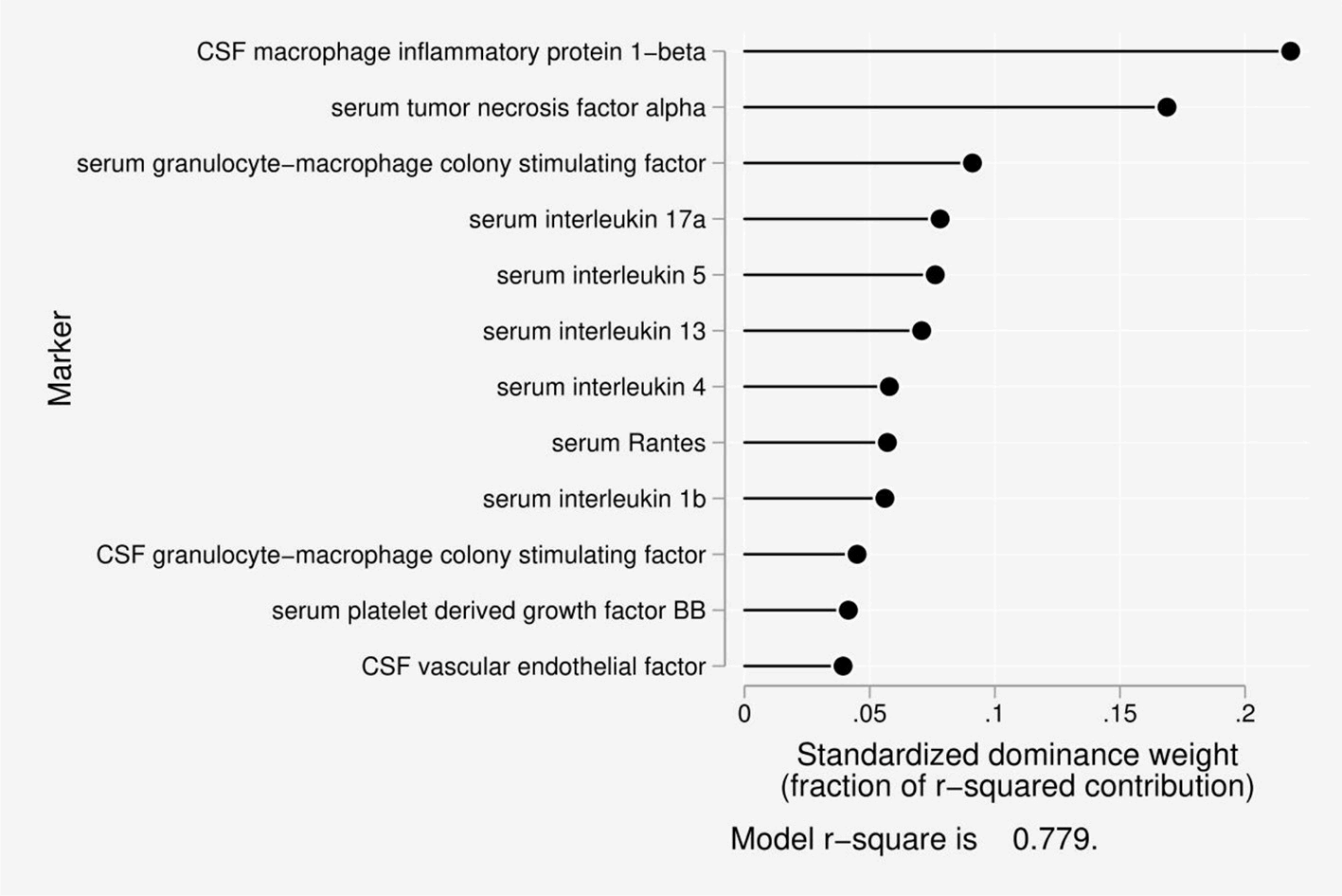
Proportionate contribution of serum and CSF factors to model r-square for BCSFB ratios for creatinine.

Twelve predictors were included in the dominance analysis for BCSFB permeability to creatinine that included estimates of 4,095 subset models. The overall model *R*^2^ was 0.78. The proportionate contribution to this model *R*^2^ is shown in Figure 2. The dominant predictor of the creatinine CSF/serum ratio was cerebrospinal fluid macrophage inflammatory protein 1-beta (MIP1β), which accounted for 22% of the overall model *R*^2^.

### Cytokine, chemokine, and trophic factors predicting BBB to albumin

**Figure 3:**
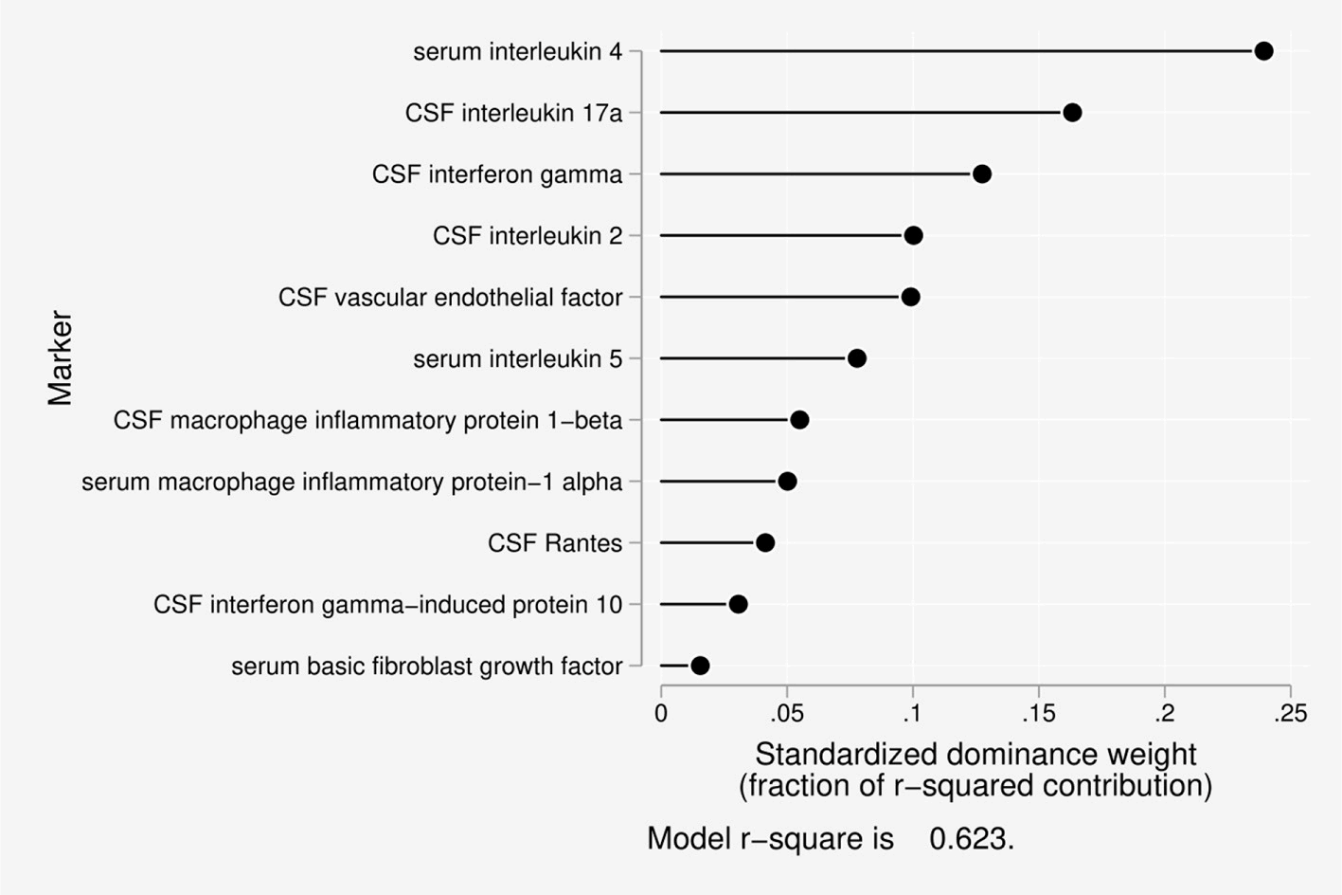
Proportionate contribution of serum and CSF factors to model r-square for BCSFB ratios for albumin.

Eleven predictors were included in the dominance analysis for the BCSFB to albumin that included estimates of 2,047 subset models. The overall model *R*^2^ was 0.62. The proportionate contribution to this model *R*^2^ is shown in Figure 3. The dominant predictor for the albumin CSF/serum ratio was serum interleukin-4 (IL-4), which accounted for 24% of the overall model *R*^2^.

Table 4 shows the physical characteristics of urea, creatinine, and albumin in comparison with the molecules most closely associated with their respective BCSFB ratios in the dominance analyses. A relationship between these ratios and molecular weight, isoelectric point, or molecular diameter of the associated molecules is not apparent.

**Table 4:** Molecular properties of serum and CSF cytokines, chemokines, and trophic factors most closely related to BCSFB ratios.

**Factor**	**Molecular weight (daltons) in humans**	**Isoelectric point (pI) in mice**	**Molecular diameter angstroms (Å)**	**Reference link**
Urea	60	8.19	3.4	http://jgp.rupress.org/content/jgp/44/6/1189.full.pdf
Monocyte chemoattractant protein 1	11,025	9.81	60.7	http://www.rcsb.org/structure/3IFD
Interleukin-15	12,000	5.16	129.2	http://www.rcsb.org/structure/4GS7
Interleukin-1 receptor antagonist	20,055	8.30	117.3	http://www.rcsb.org/structure/1ILR
Interleukin-2	15,000	4.88	79.2	http://www.rcsb.org/structure/1M4C
Creatinine	113	11.19	6.0	https://www.ncbi.nlm.nih.gov/pubmed/2418254
Macrophage inflammatory protein 1 beta	10,166	8.88	186.6	http://www.rcsb.org/structure/2X6L
Tumor necrosis factor-alpha	17,400	5.01	117.0	http://www.rcsb.org/structure/1TNF
Albumin	66,437	4.90	58.4	https://www.ncbi.nlm.nih.gov/pubmed/11449540#
Interleukin-4	15,000	9.17	90.6	http://www.rcsb.org/structure/2B8U
Interleukin-17a	35,000	5.06	119.9	http://www.rcsb.org/structure/4HR9
Interferon-gamma	17,000	8.72	315.0	http://www.rcsb.org/structure/1HIG
Interleukin-2	15,000	4.88	79.2	http://www.rcsb.org/structure/1M4C
Vascular endothelial growth factor	38,200	9.27	95.7	http://www.rcsb.org/structure/2XV7

## Discussion

Neuroinflammation has been widely documented in the AD brain (Zhang and Jiang, 2015; Bagyinszky et al., 2017; McManus and Heneka, 2017); however, the mechanisms whereby inflammatory events and mediators contribute to AD pathogenesis have not been clearly defined. A recent study demonstrated that pro-inflammatory proteins of importance in AD include interleukin 1 alpha, tumor necrosis factor, and complement C1q, secreted by activated microglia, which induce a reactive form of neurotoxic astrocyte that cannot support neuronal survival, outgrowth, synaptogenesis and phagocytosis, leading to the death of neurons and oligodendrocytes (Liddelow et al., 2017). Similarly, BBB dysfunction in AD is an early and consistent feature of the disease (Bell and Zlokovic, 2009), although the drivers of this pathologic process are also not known. Our study adds to growing evidence suggesting that inflammation plays a role in the pathogenesis of altered BCSFB function in early AD.

One previous study demonstrated that thrombin and high-mobility group box protein 1 (HMGB1) are proximate proinflammatory mediators of BBB dysfunction in MCI patients as well as in AD (Festoff et al., 2016). Interestingly, serum HMGB1 levels in that study were significantly elevated in MCI patients compared to controls or AD patients, raising the question as to whether systemic inflammation may have its greatest effects at this early stage of disease. Along these lines, we previously found that BCSFB disruption was greater in MCI than in AD, suggesting that this phenomenon is particularly important at the early stage of disease (Johanson et al., 2018). These data highlight the important issues of timing (early vs. later) BCSFB changes, as well as the relationship between processes of peripheral and brain-derived inflammation.

Our data demonstrate specific inflammatory mediators (an inflammatory profile) are associated with different degrees of BCSFB disruption. Furthermore, the data show that the apparent evolution of BCSFB changes from increased permeability to smaller probes like urea to more pronounced BCSFB perturbation to a larger molecule, albumin, is correlated with a relative shift of inflammatory factors from serum to CSF. While changes in CSF cytokine levels may reflect transport and distribution of those molecules between blood and CSF, another possibility is that in MCI and AD there is enhanced penetration of leukocytes from blood to CSF, e.g., across the choroid plexus, and that those leukocytes that have accessed CSF are now being stimulated to secrete cytokines into the CSF.

Differences in how inflammation patterns in serum and CSF relate to small, then later large BBB permeability changes may shed light on disease pathogenesis and could also be useful as a biomarker to monitor progression. The idea that an inflammatory profile approach is useful to classify AD is supported by previous work which demonstrated that an algorithm comprised of 21 serum inflammatory proteins from 150 AD cases and 150 controls yielded sensitivity and specificity of 0.90 for correctly classifying AD (O'Bryant et al., 2014). Other studies using a meta-analysis of cytokines in AD also identified a specific pattern of cytokines (IL-6, TNF-α, IL-1β, TGF-β, IL-12, and IL-18) in serum as well as in CSF (TGF-β) (Swardfager et al., 2010).

In our current study, disruption of the BCSFB to urea was most significantly related to monocyte chemoattractant protein-1 in serum, followed by interleukin (IL)-15, IL-1rα, and IL-2 in serum. Monocyte chemoattractant protein-1 is a chemokine anchored in the plasma membrane that is secreted by monocytes, macrophages and dendritic cells. Induced in astrocytes by PDGF-BB, it attracts monocytes promoting their transmigration through a disrupted blood-brain barrier. Increased levels impair attention; executive function, and psychomotor speed (Bethel-Brown et al., 2012). IL-15 is a pleiotropic pro-inflammatory cytokine that is produced by activated monocytes, macrophages, and dendritic cells. It promotes T cell proliferation and cytotoxicity via natural killer and cytotoxic T cells. It is also produced by activated astrocytes, and elevated serum levels occur in AD (Bishnoi et al., 2015). IL-RA increases adhesion molecule expression and induces metalloproteinases and prostaglandins. It serves a neuroprotective role as an inhibitor of cytotoxic, ischemic, excitotoxic, and traumatic injury in the brain (Simi et al., 2007). IL-2 is a cytokine signaling regulator of activities in leukocytes responsible for immunity. It increases T cell proliferation and activates B Cells. It serves a neuroprotective role in maintaining septohippocampal cholinergic neurons (Meola et al., 2013).

Disruption of the BCSFB to creatinine, a larger molecule governed by active transport among CNS compartments, was related to monocyte inhibitor protein-1b in CSF as the dominant predictor, followed by tumor necrosis factor-α in serum. Monocyte inhibitor protein-1b is a chemokine with chemoattraction for NK and T cells. It induces synthesis and release of pro-inflammatory cytokines from fibroblasts and macrophages. It has been associated with impaired attention, executive function, and psychomotor speed, and it shows increased expression with oncornavirus induced spongiform neurodegeneration (Askovic et al., 2001). Tumor necrosis factor-α is a pro-inflammatory cytokine of activated macrophages. It induces expression of other cytokines, chemokines, metalloproteinases, and adhesion molecules in acute phase responses. It causes fever, cachexia, inflammation, and apoptosis. Dysregulated expression is seen in neurodegeneration including AD, and in major depression. In neurodegeneration, TNF-α induces neuronal excitotoxic injury (via glutamate), accumulates around senile plaques, and it can also increase synaptic transmission (Spittau et al., 2012; Ramesh et al., 2013; Zheng et al., 2016).

Disruption of the BCSFB to albumin, a much larger molecule, was most significantly related to IL-4 in serum, followed by IL-17a, interferon-gamma, IL-2, and vascular endothelial growth factor (VEGF) in CSF. IL-4 is a cytokine that induces differentiation of naïve T cells and regulates humoral and adaptive immune responses. It's anti-inflammatory actions reduce thymus-1 type cytokine, IFN-γ, macrophages, and dendritic cell IL-12. IL-4 is potentially neuroprotective for cortical neurons by modulating excitability (Mori et al., 2016). IL-17a is a pro-inflammatory cytokine produced by T helper cells and induced by IL-23. It recruits monocytes and neutrophils to sites of inflammation. It plays a role in auto-immune diseases and microbial defenses as well as T-cell mediated delayed-phase inflammatory injury in ischemic stroke (Swardfager et al., 2013). Interferon-gamma is a pro-inflammatory cytokine and potent activator of macrophages. It plays a role in mediating innate and adaptive immune responses, as well as delayed immune response. Interferon-gamma mediates delayed post-ischemia neurodegeneration via IFN-γ secreted by splenic macrophages, and it promotes inflammatory mediated impairment of neural stem and neuroprogenitor cell maturation and differentiation (Walter et al., 2011; Walter and Dihne, 2012; Seifert and Pennypacker, 2014; Seifert et al., 2014). IL-2 is a neuroprotective cytokine (Meola et al., 2013). CSF levels of VEGF are elevated in normal brain aging. VEGF is a trophic factor that stimulates *de novo* vasculogenesis and angiogenesis, fibroblast proliferation, and monocyte/macrophage migration. It restores oxygen supply to tissues injured by deprivation and increases microvascular permeability and may be neuroprotective, as reduced CSF levels correlate with hippocampal atrophy, loss of executive functions and memory. It also has an interactive effect with Aβ1-42 (Hohman et al., 2015).

It is well established that inflammatory cytokines can contribute to BBB dysfunction in human disease (Coisne and Engelhardt, 2011; Elwood et al., 2017), including studies of the BCSFB using albumin ratios in meningitis showing relationship to TNF- α (Sharief et al., 1992), and cardiac surgery showing relationship to IL-6 and IL-8 (Reinsfelt et al., 2012). In a study of 141 patients with probable AD, significant correlation was reported between levels of CSF anti-chymotrypsin, oxidized low-density lipoprotein and the ratio of CSF to serum albumin (Sun et al., 2003). More recently, in another of 45 AD patients, 18 MCI subjects, and 23 non-demented controls, elevated concentrations of YKL-40, a marker of glial inflammation, correlated significantly with increased albumin ratio and decreased Abeta42/40 ratio in AD patients (Muszynski et al., 2017).

Unlike previous reports, rather than looking at individual cytokines, we have looked at multiple cytokines simultaneously using a relatively new analytic approach, providing new insights about the nature of inflammatory cascades on both sides of the barrier between blood and CSF. Furthermore, we are the first to associate these barrier changes with inflammation for three different molecules (urea, creatinine and albumin), one of which (creatinine) uniquely relates to transporter disruption. Taken together with previous observations, the results of our study suggest that changes in BCSFB function resulting in altered permeability and transport are related to expression of specific inflammatory proteins, and that the complex shifting distribution of these proteins from serum to CSF in AD and MCI is correlated with more severe perturbations in BCSFB function. We did not observe a simple relationship between the physicochemical characteristics of urea, creatinine, and albumin and the cytokines most related to alterations in their BCSFB ratios, arguing somewhat in favor of the cytokines playing a causative role in BCSFB dysfunction, rather than simply being a physical marker of BCSFB sieve characteristics.

There are limitations to the current study as well as other valid interpretations of the data. BCSFB permeability and transport perturbation and expression of inflammatory markers may be linked but not causally related. In this regard, in a mouse model of epilepsy, a higher concentration of chemokines and pro-inflammatory cytokines in the serum and higher expression in activated microglia were documented without BBB disruption (Okuneva et al., 2016). However, that study assessed BBB changes only in relation to the large molecule, albumin.

It is also difficult to assess the effects of peripheral inflammation as distinct and separately arising from inflammatory changes in the brain. A growing literature documents the influence of systemic inflammatory, oxidative and immune processes on biochemical events in the brain (Yu et al., 2016; Main and Minter, 2017; Mietelska-Porowska and Wojda, 2017). Peripheral administration of the soluble TNF inhibitor XPro1595 modifies brain immune cell profiles, decreases beta-amyloid plaque load, and rescues impaired long-term potentiation in 5xFAD mice (MacPherson et al., 2017). We have previously shown that systemic oxidative stress correlates with expression of inflammatory proteins in the cerebral vasculature and impaired cognition in ApoE knockout mice (Evola et al., 2010).

An additional limitation is our small sample size and the relatively high number of comparisons. Small sample size studies are at greater risk of both type-I errors and type-II errors than what would be expected given the nominal significance levels used in forming hypothesis tests (Button et al., 2013). To address this, we relied upon descriptive effect size statistics, reported confidence intervals for effect size statistics, and used Bonferroni correction to significance level. These procedures, combined with the observation that the probability of finding 13 of 54 independent contrasts with absolute value of a standardized effect size greater than 1.0 is less than 1 in 1000, add confidence to our interpretation that at least some of our findings are unlikely to reflect random noise.

Of note, it is possible that the albumin ratio may not accurately reflect the degree of BBB breakdown. Recent studies suggest that albumin levels in the CSF can be affected by many factors which include uptake of extravasated albumin by microglia, astrocytes (Ivens et al., 2007; LeVine, 2016), neurons and NG2 positive cells (Braganza et al., 2012). A strength of our study was that we also included ratio measurements of urea and creatinine for comparison with the data derived from albumin measurements.

We did not have the ApoE genetic status on most patients. This information was also unavailable in our control subjects. Since ApoE may have a modulating effect on these relationships (Zipser et al., 2007; Donahue and Johanson, 2008; Halliday et al., 2016), future research should address the role of ApoE combined with inflammation on BBB disruption in AD. The number of subjects we examined was small, limiting our ability to analyze relationships between disease severity and differences in inflammatory effects on the BCSFB in MCI and AD. Furthermore, though we did include cognitively normal subjects, we did not differentiate those with possible preclinical stage AD using biomarkers and brain imaging techniques.

Lastly, while we made efforts to exclude subjects with active inflammatory and infectious disease, the presence of minor inflammation producing illness in some subjects, particularly those drawn from the emergency department, could have influenced our results. We also did not routinely collect details on the subjects regarding various vascular risk factors for dementia like hypertension and diabetes, so future studies in this area should control for such factors, particularly with regard to blood cytokines.

## Conclusion

In sum, our report should be regarded as preliminary evidence suggesting the possibility that systemic and central neuroinflammatory processes produce BCSFB disruption early in the course of AD. Such damage to the BBB may result in a brain that is highly vulnerable to inward leakage of neurotoxins and weakening of important energy systems dependent on active transport of nutrients such as creatine from the systemic circulation that in turn could lead to or hasten neurodegeneration. Future research should examine whether such processes occur during the preclinical stage of the disease when anti-inflammatory and BBB neuroprotective therapies may have their greatest impact on the prevention of dementia. While our study is essentially exploratory, the unique data on BCFB permeability in relation to cytokines and chemokines should prompt new efforts to experimentally focus on these factors and their mechanisms of barrier impairment, in relation to the changing CSF environment in MCI and AD.

## Disclosure

BO reports having received research funding from Biogen, Lilly, Avid, Merck, Janssen, AbbVie, and Long Term Care Group, as well as consulting fees from Amgen.

## Author contributions

BO, RJ, LD, SdlM, ES, CJ, CD, and PG contributed to the conception or design of the work; or the acquisition, analysis, or interpretation of data for the work; and drafting the work or revising it critically for important intellectual content; and final approval of the version to be published; and agreement to be accountable for all aspects of the work in ensuring that questions related to the accuracy or integrity of any part of the work are appropriately investigated and resolved.

### Conflict of interest statement

The authors declare that the research was conducted in the absence of any commercial or financial relationships that could be construed as a potential conflict of interest.
